# Malpositioned orbital implants: indications for implant removal and secondary reconstructive techniques

**DOI:** 10.3389/fsurg.2026.1878546

**Published:** 2026-07-17

**Authors:** Sara M. Hussein, Basel A. Sharaf, Krishna Sinha, Andrew J. Moyo, Jonathan M. Morris, Lilly H. Wagner

**Affiliations:** 1 Division of Plastic and Reconstructive Surgery, Department of Surgery, Mayo Clinic, Rochester, MN, United States; 2 Mayo Clinic Alix School of Medicine, Phoenix, AZ, United States; 3 Anatomic Modeling Lab, Department of Radiology, Mayo Clinic, Rochester, MN, United States; 4 Division of Oculoplastic and Orbital Surgery, Department of Ophthalmology, Mayo Clinic, Rochester, MN, United States

**Keywords:** computer-assisted surgery, diplopia, imaging, three-dimensional, orbital fractures, orbital implants, postoperative complications, reoperation

## Abstract

**Background:**

Malpositioned orbital implants are an underappreciated source of significant postoperative morbidity, often necessitating revision surgery. Decision-making regarding practical frameworks to guide revision remains challenging and is not well standardized or characterized.

**Objective:**

To analyze clinical presentations, radiographic failure patterns, multidisciplinary surgical strategies, and outcomes of patients undergoing revision surgery for malpositioned orbital implants.

**Methods:**

A single-center retrospective consecutive case series was conducted of patients who underwent removal or replacement of malpositioned orbital implants. Patient demographics, presenting symptoms, radiographic findings, surgical approaches, implant management strategies, and postoperative outcomes were reviewed. Most cases were co-managed by plastic/reconstructive and oculoplastic surgeons. Descriptive statistics were used to summarize findings.

**Results:**

Five of seven patients (71.4%) were female, with a median age of 60 years (range 29–78). Diplopia was the most common presenting symptom (5/7, 71.4%) along with pain or swelling (5/7, 71.4%) followed by globe malposition (3/7, 42.9%). CT demonstrated implant protrusion into orbital soft tissue in 4/7 patients (57.1%) and peri-implant inflammatory collections in 3/7 (42.9%). Out of our 7 revision cases, four patients (57.1%) underwent explantation alone, and three (42.9%) underwent secondary reconstruction with custom or semi-custom implants. At follow-up, diplopia improved or resolved in 4/5 affected patients (80%), and pain, swelling, and globe position improved in all symptomatic patients.

**Conclusion:**

Malpositioned orbital implants demonstrate heterogeneous failure mechanisms that require individualized surgical management. A multidisciplinary approach can guide decision-making regarding implant removal versus revision of reconstruction, enabling safe revision surgery and favorable clinical outcomes. Additionally, virtual surgical planning and intraoperative navigation are enablers of safe and precise revision orbital surgery.

## Introduction

Orbital fractures are among the most common injuries encountered in craniomaxillofacial trauma, and surgical reconstruction with implants is the standard of care for displaced fractures causing functional deficits or significant volume loss (1). Despite refinements in surgical technique and implant technology, implant malposition remains a clinically significant complication. A multicenter analysis of 232 primary orbital reconstructions found an overall revision rate of 6.5%, rising to 14% for complex midfacial fractures with rim involvement (2). The risk of malposition increases substantially with fracture complexity; Schlittler et al. reported that 23% of titanium mesh implants demonstrated poor positioning, with all five patients requiring revision having multiple-wall fractures, and the major reason for revision being a defect too large for the prescribed plate and with 17% ultimately requiring revision surgery (1). The late-onset complications, including heterotopic bone formation, cystic degeneration, and chronic inflammation, are likely underestimated and may not manifest for years after initial repair (3–5).

Malpositioned orbital implant outcomes extend beyond cosmesis and may result in a wide spectrum of complications, including persistent diplopia, ocular motility restriction, pain, swelling, globe malposition, sensory disturbances, and optic nerve injury (6). These problems may arise from improper implant sizing, non-anatomical contour, inaccurate placement, migration, or direct contact with critical orbital structures such as extraocular muscles or neurovascular bundles. The management of failed orbital implants is complex and requires individualized surgical decision-making. Radiographic evaluation, particularly computed tomography (CT) and magnetic resonance imaging (MRI), plays a critical role in identifying implant-related failure patterns, yet the relationship between specific imaging findings and clinical symptoms is incompletely characterized (1, 6).

While plates and screws used in facial fracture reconstruction demonstrate variable long-term outcomes, with removal rates ranging from 7% to 12% depending on location and patient factors (7), the specific criteria for orbital implant revision remain poorly defined. Management options range from implant repositioning or selective trimming to complete explantation with or without secondary reconstruction. Existing literature has largely focused on outcomes following primary orbital reconstruction or on isolated complications, with limited data on indications for implant removal and individualized surgical approaches for revisions (8, 9).

Despite a growing literature on primary orbital reconstruction outcomes and on isolated complications such as cyst formation or implant migration, the specific question of when to remove a malpositioned implant, when to replace it, and when to perform revisions with a patient-specific implant (PSIs) remains poorly defined. The present study aims to fill this gap by correlating patient-reported symptoms, radiographic patterns, surgical approaches, and postoperative outcomes across a consecutive case series managed collaboratively by plastic surgery and oculoplastic surgery, with comprehensive documentation to facilitate surgical education and decision-making.

## Methods

### Study design and patient population

A retrospective case series was conducted at a single tertiary academic center following institutional review board approval (IRB #: 23-010055), and reported in accordance with the STROBE statement for observational studies (10). The records of all patients who underwent surgical intervention for malpositioned orbital implants over a five-year period were reviewed. The cohort was identified from a prospectively maintained departmental operative database. Inclusion required: (1) History of orbital fracture repair or orbital implant placement, (2) Subsequent surgical intervention for implant malposition or implant-related symptoms, and (3) Complete implant removal, implant replacement, or secondary orbital reconstruction. Patients undergoing revision surgery for acute trauma, oncologic reconstruction, or non-implant-related orbital pathology were excluded. Based on existing clinical records, the original surgical approach used at the index repair (transconjunctival pre-septal vs. post-septal, subciliary, subtarsal, or transcaruncular) was incomplete in most of the cases (5 out of 7). When available, the surgical approach used at the index primary repair was abstracted from prior operative reports.

Demographics and baseline characteristics were collected, including age at revision surgery, sex, weight, height, body mass index (BMI), smoking status (current, former, or never), and relevant medical comorbidities. Operative data included anesthesia type, American Society of Anesthesiologists (ASA) classification, anesthesia duration and operative times, estimated blood loss, and surgical approach.

### Indications of revision surgery

**Figure 1:**
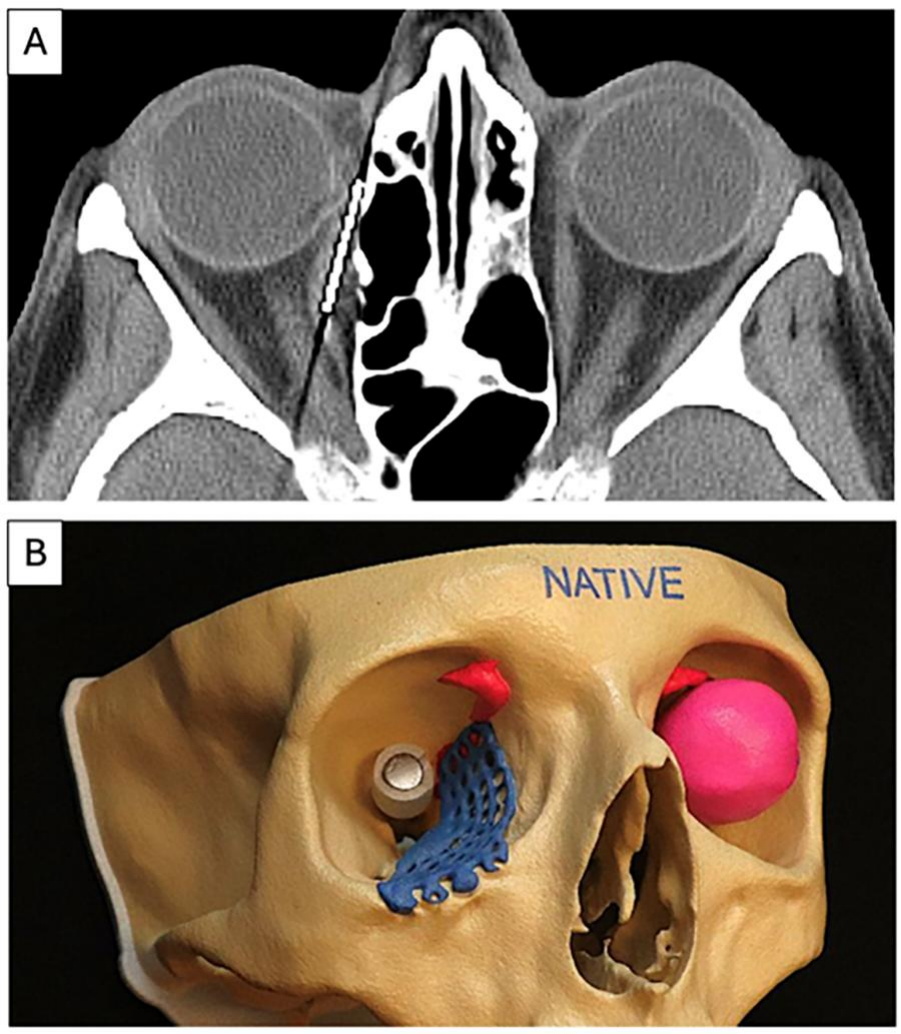
3D printed model to improve safety of hardware removal. **(A)** Axial soft-tissue CT shows direct contact of the implant with the medial rectus muscle and proximity of the implant edge to the optic nerve. **(B)** 3D printed model displays the malpositioned orbital implant and its relationship to the globe, optic nerve and superior oblique muscle (globe model was removed from magnetic attachment); surgeons utilize this model for planning of safest vectors for implant mobilization and removal.

**Figure 2:**
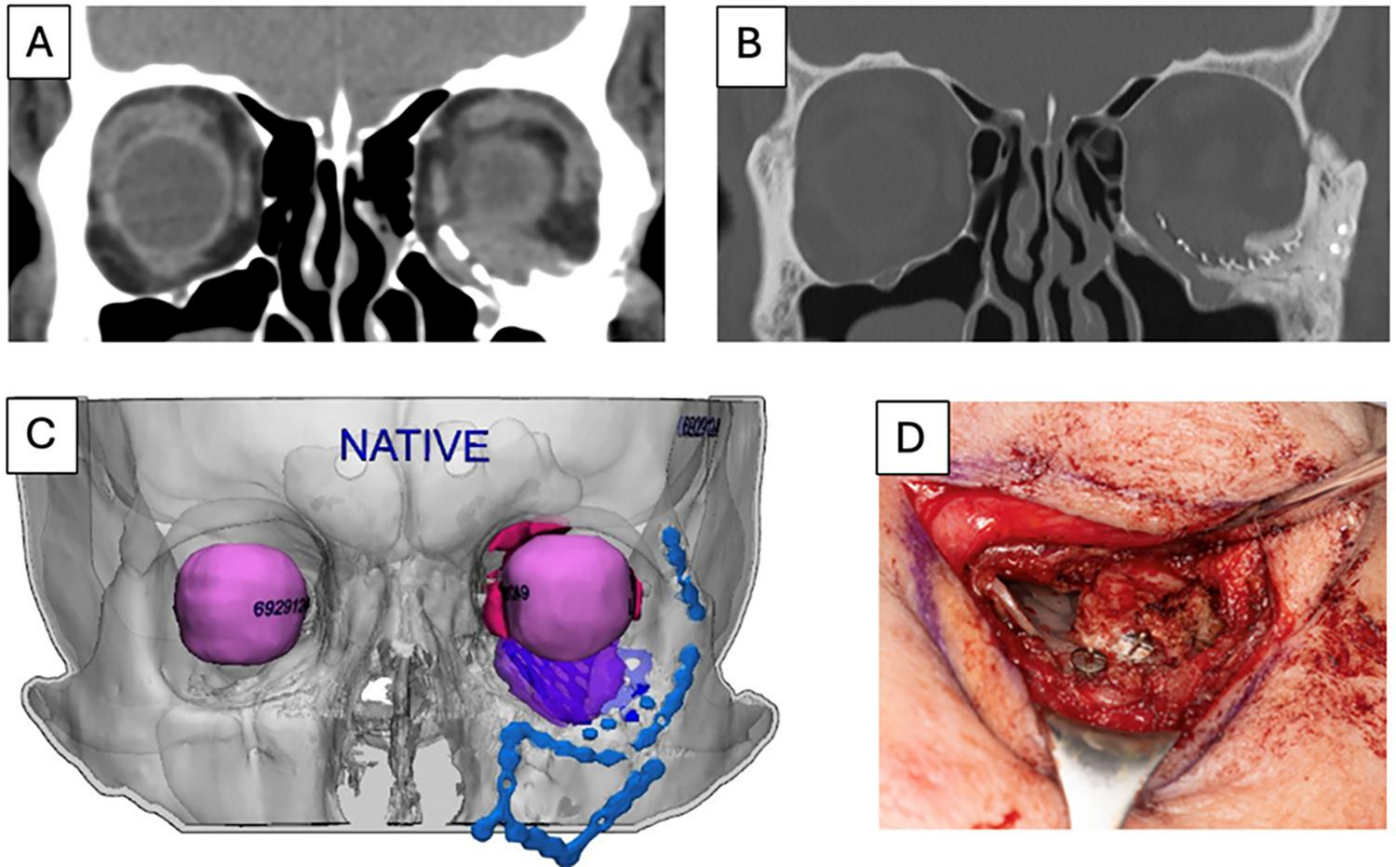
Virtual surgical planning for removal of hardware and capsule. **(A)** Soft-tissue window coronal CT demonstrates presence of a large capsule and hardware malposition with protrusion into orbital soft tissue. **(B)** Bone window coronal CT shows heterotopic bone formation surrounding the implant. **(C)** Digital model displays mass effect of capsule on the globe and precise location of fixation screws. **(D)** Intraoperative view of capsule, aberrant bone and hardware; the implant and abnormal capsular tissue were removed and no additional hardware was placed, because support for the globe was deemed adequate.

**Figure 3:**
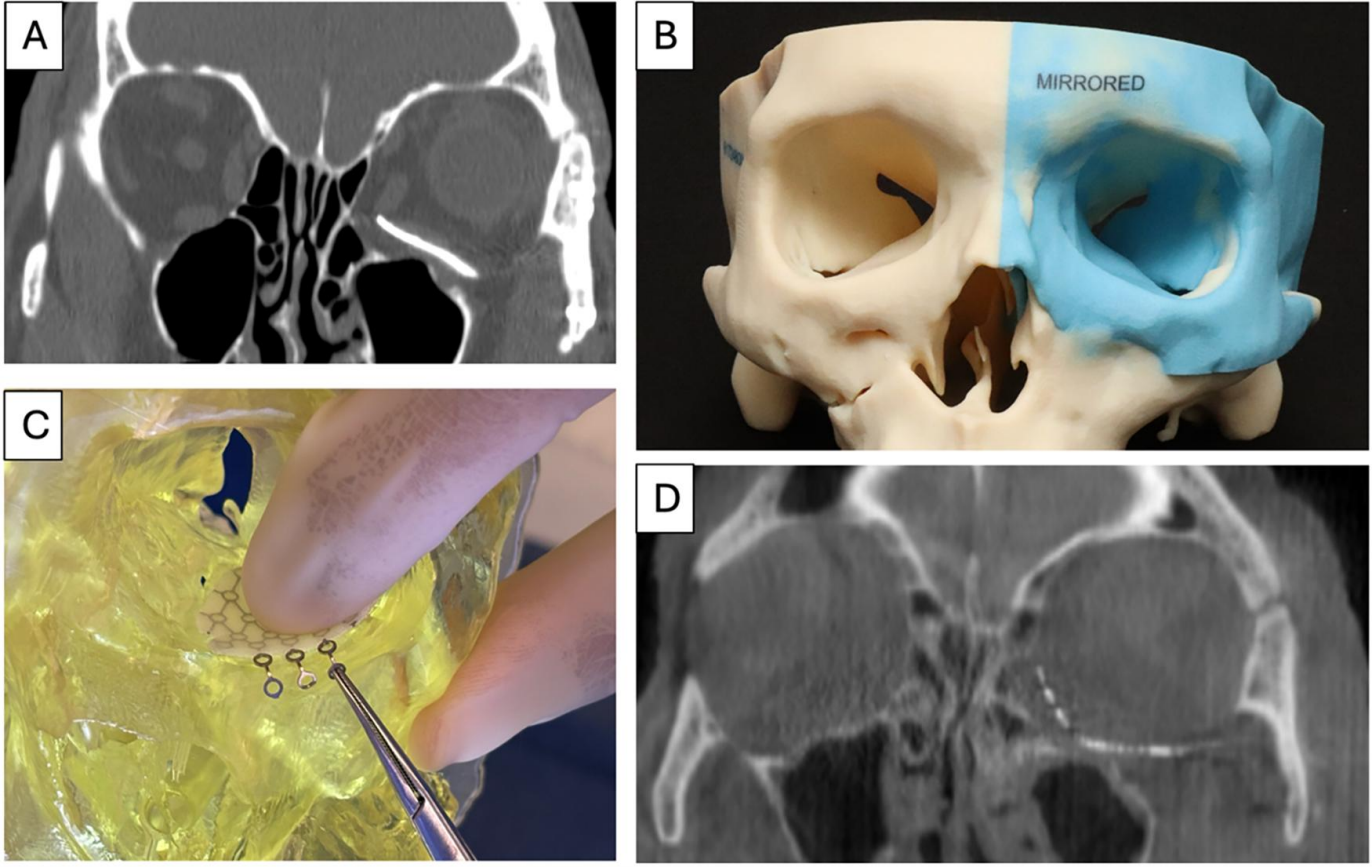
Correction of undersized hardware using 3D models. **(A)** Previously placed implant is not fully correcting the large orbital floor and medial wall fracture; the patient had 5-mm residual enophthalmos. **(B)** 3D model with mirror-image overlay of unaffected right side onto the left orbit. **(C)** Intraoperative shaping and molding of standard MEDPOR titan orbital floor implant on a sterilized mirror-image model. **(D)** Intraoperative CT demonstrates adequate implant placement and correction of orbital deformity with restored symmetry.

Patient-reported symptoms of prompting revision were documented, including diplopia, pain, discomfort, swelling, and visible deformity. Preoperative CT/MRI imaging was reviewed to identify radiographic failure patterns, including implant protrusion into extraconal orbital soft tissue, intraconal implant position, contact with extraocular muscles or optic nerve, implant over- or undersizing, asymmetric orbital wall contour, and implant-associated fluid collections. All cases were managed using a coordinated multidisciplinary approach involving plastic and reconstructive surgery and oculoplastic surgery. Operative planning was performed jointly, incorporating detailed review of preoperative CT/MRI review, virtual surgical planning (VSP) with mirror-image overlay, 3D-printed patient-specific anatomical models, and intraoperative navigation using the Medtronic StealthStation™ platform in selected complex cases (Figures 1–3). Surgical responsibilities were shared, with oculoplastic surgeons leading exposure, protection of ocular structures, and eyelid/canthal reconstruction and plastic surgeons leading hardware mobilization, removal, bony work, and secondary fracture reconstruction; both attending surgeons were co-scrubbed throughout due to the complexity of these cases.

Importantly, pre- and postoperative ocular motility, globe position, and eyelid metrics were assessed by oculoplastic surgery and orthoptic technicians as part of routine perioperative care. The assessment included (a) examination of alignment in primary, cardinal, and oblique gaze positions with documentation of restrictions and head-position compensation; (b) binocular single vision (BSV) field testing with qualitative reporting of the gaze positions; (c) Hertel exophthalmometry recorded as right/left   ×   base (mm); (d) margin reflex distances MRD1 and MRD2 (right/left, mm); and (e) intraoperative forced duction testing of all four rectus muscles at the start and end of each revision procedure, and (f) full orthoptic examination with quantification of misalignment, recorded in prism diopters, where strabismus was present. Pre-operative assessments were obtained at the most recent oculoplastic visit before revision; post-operative assessments were obtained at the most recent follow-up clinic visit. Hess screen testing was not uniformly available across the cohort and is not reported.

#### Surgical technique

A transconjunctival inferior orbitotomy was the primary approach in most cases (71.4%), frequently augmented with transcaruncular extension for medial wall exposure and lateral canthotomy/cantholysis to improve access. In selected cases, subciliary, subtarsal, lateral brow, transfacial, or transoral approaches were utilized based on implant location, prior scars, and associated facial hardware. Case 3 required the most complex multi-approach strategy, a combination of transoral (hemi-LeFort 1), transfacial, lateral brow, and transconjunctival incisions, to address symptomatic hardware across multiple facial subunits. Case 5 was approached through the previous surgery scar through the lower lid. Case 6 was also approached through a horizontal lower lid incision specifically designed to simultaneously excise the cutaneous fistula tract.

Following local infiltration with 1.5% lidocaine and 1:100,000 epinephrine, the lower eyelid was retracted by a Desmarres retractor, and a preseptal transconjunctival dissection was carried to the inferior orbital rim. The periosteum was incised to expose the implant capsule (Figure 5). Careful blunt and sharp dissection was performed along the superior and inferior surfaces of the existing orbital implants using Freer elevators and malleable retractors, with meticulous attention to protecting the globe, extraocular muscles, lacrimal apparatus, and optic nerve. Hemostasis was maintained with judicious application of monopolar and bipolar cautery, avoiding cautery use near the optic nerve, and application of hemostatic and vasoconstrictive agents, again avoiding placement near the optic nerve and avoiding use of expanding matrices such as gelatin sponges (e.g., Gelfoam) near the orbital apex. Forced duction testing was performed intraoperatively to assess for muscle entrapment or tethering.

### Outcomes and follow-up

Primary outcomes included resolution or improvement of diplopia, pain, swelling, periorbital asymmetry, globe position, and sensory disturbances. Secondary outcomes included complications (infraorbital nerve hypoesthesia, infection, and immediate postoperative bleeding), need for additional surgical procedures, and time to last follow-up. Diplopia outcomes were assessed at each postoperative clinic visit based on patient-reported symptoms and clinical examination.

### Statistical analysis

Descriptive statistics were used to summarize all variables. Continuous variables are reported as mean ± standard deviation or median with interquartile range, and categorical variables as frequencies and percentages. Given the exploratory nature of this case series, inferential statistical testing was not performed.

## Results

### Patient characteristics

Seven patients underwent revision surgery for malpositioned orbital implants over the study period (Table 1). Five patients were female (71.4%), and two were male (28.6%). Median age at revision was 60 years (range 29–78 years). The median interval from primary fracture repair to revision surgery was 26 months (range 1–144 months), underscoring the heterogeneous, often longdelayed nature of orbital implant failure. Laterality was left-sided in five patients and right-sided in two. All procedures were performed under general anesthesia. ASA classification ranged from I to III (ASA I: *n* = 1; ASA II: *n* = 4; ASA III: *n* = 2), reflecting the diverse comorbidity burden of this patient population. The mean BMI was 24.4 ± 4.0 kg/m². Operative time ranged from 50 min to 5 h 46 min, reflecting marked variability in procedural complexity. Estimated blood loss was minimal across all cases (range 5–75 mL). Median follow-up from revision surgery to last clinic visit was 2.2 months (range 1.1–16.4 months).

**Table 1:** Patient demographics and baseline characteristics.

Case	Sex	Age at Surgery	Side	BMI	ASA	Comorbidities	DM	HTN	Smoking
1	F	29	Left	22.5	1	—	No	No	_
2	F	47	Left	25.7	2	—	No	No	_
3	M	60	Left	27.6	3	—	No	No	_
4	F	61	Right	18.2	2	—	No	No	Former
5	M	43	Left	30.5	2	Bilateral glaucoma	No	Yes	Former
6	F	78	Left	20.4	3	cardiac arrhythmia; COPD; dyslipidemia; heart failure hypertension; hypothyroidism; peripheral neuropathy;	No	Yes	None
7	F	67	Right	25.5	2	—	No	No	Current

### Indications for revision surgery

All patients were symptomatic at presentation (Table 2). The most frequent clinical indications prompting revision included diplopia (5/7 patients, 71.4%), followed by concurrent pain, discomfort, or recurrent swelling (71.4%) and enophthalmos or abnormal globe position (3/7, 42.9%). One patient presented with chronic infectious or inflammatory complications: Case 6 with cellulitis and cutaneous fistula formation. Diplopia was predominantly vertical in nature and, in several cases, position-dependent or associated with torsional components. Thus, Case 1 demonstrated hypertropia in upgaze, Case 2 showed hypotropia attributable to inferior rectus tethering by the displaced implant, and Case 4 exhibited vertical diplopia with a torsional component and limitation of supra- and infraduction, likely caused by restriction of the inferior oblique muscle. In two patients, diplopia was absent in primary gaze but present in upgaze or downgaze, highlighting subtle but functionally significant motility disturbances (Supplementary Table S1).

**Table 2:** Clinical indications and radiographic failure patterns.

Case	Trauma Mechanism	Original Fracture Type	Accompanying Fractures	Patient Symptoms	Radiographic Findings
1	Sports (softball)	Orbital medial wall	—	Diplopia (hypertropia on upgaze, vertical; no primary gaze diplopia); pain; discomfort; swelling; enophthalmos. Hertel 16/15 × 97; MRD1 4/4; MRD2 5/6. Recurrent monthly periorbital ecchymosis & pain since Feb 2023 (dog bump). Managed with antibiotics to prevent orbital infection.	Proximity to optic nerve (orbital apex); supraorbital/supratrochlear/inferior trochlear vessels (superonasal orbit); screw possibly exposed to maxillary sinus. Protrusion into orbital soft tissue.
2	MVA (ejection; car vs. cow)	Orbital floor (ZMC, minimally displaced left)	Minimally displaced left ZMC & NOE; nondisplaced mandibular body fracture; coronoid fracture of left mandible	Vertical binocular diplopia; pain; swelling; enophthalmos; hypo-globus. Hertel 19/15 × 101.	Left enophthalmos and hypoglobus. Hardware infection (ZM plate). Intraconal position/contact with extraocular muscles.
3	MVA	Orbital floor (left ZMF)	Right frontonasomaxillary fracture; right lateral brow foreign body	Pain. No documented diplopia or enophthalmos preoperatively.	—
4	Falls	Orbital roof; orbital lateral wall		Vertical diplopia (with torsional component on downgaze); Cicatricial ectropion; right lateral canthal webbing.	Inadequate size/asymmetry of implant. Medpor titanium implant slightly lifted off orbital floor; not entering intraconal space; no significant rounding of inferior rectus muscle.
5	Unspecified	Orbital medial wall	—	Diplopia; discomfort/swelling. Left hypertropia 55 prism diopters preoperatively.	Fluid-filled lesion, granulation tissue, and newly formed bone sheet above old Medpor Titan plate. Inadequate size/asymmetry.
6	Falls	Orbital floor; orbital lateral wall	—	Pain/discomfort; swelling; cellulitis with resistant organisms. Fistula tract (7 mm from lash line) in left lower lid.	Purulent material draining from capsule around hardware. No bacterial growth from evacuated fluid on culture.
7	Sports	Orbital floor; orbital lateral wall	—	Vertical diplopia; discomfort.	Medpor titanium floor implant slightly lifted off orbital floor; not entering intraconal space. Persistent abnormal signal indicative of inflammation surrounding plate.

### Radiographic orbital implant failure patterns

Preoperative imaging identified distinct, case-specific failure patterns in all patients (Table 2). Implant protrusion into extraconal orbital soft tissue was the most common finding, present in 4 of 7 patients (57.1%). In three of these cases, imaging demonstrated direct contact between the implant and extraocular muscles (Figure 1), with one patient (Case 1) showing additional proximity to the optic nerve at the orbital apex, a high-risk configuration demanding meticulous dissection planning. Peri-implant inflammatory collections, capsule formation, or infectious fluid were identified in three patients (42.9%; Figure 2). Case 5 exhibited a large fluid-filled capsule, extensive granulation tissue, and a continuous sheet of heterotopic bone overlying the implant, all confirmed histopathologically as pyogenic granuloma with reactive bone formation. Implant undersizing or volumetric asymmetry was the primary finding in two patients (Cases 4 and 7), manifesting as residual enophthalmos and symptomatic globe malposition (Figure 3).

### Multidisciplinary approach, and intraoperative findings

**Figure 4:**
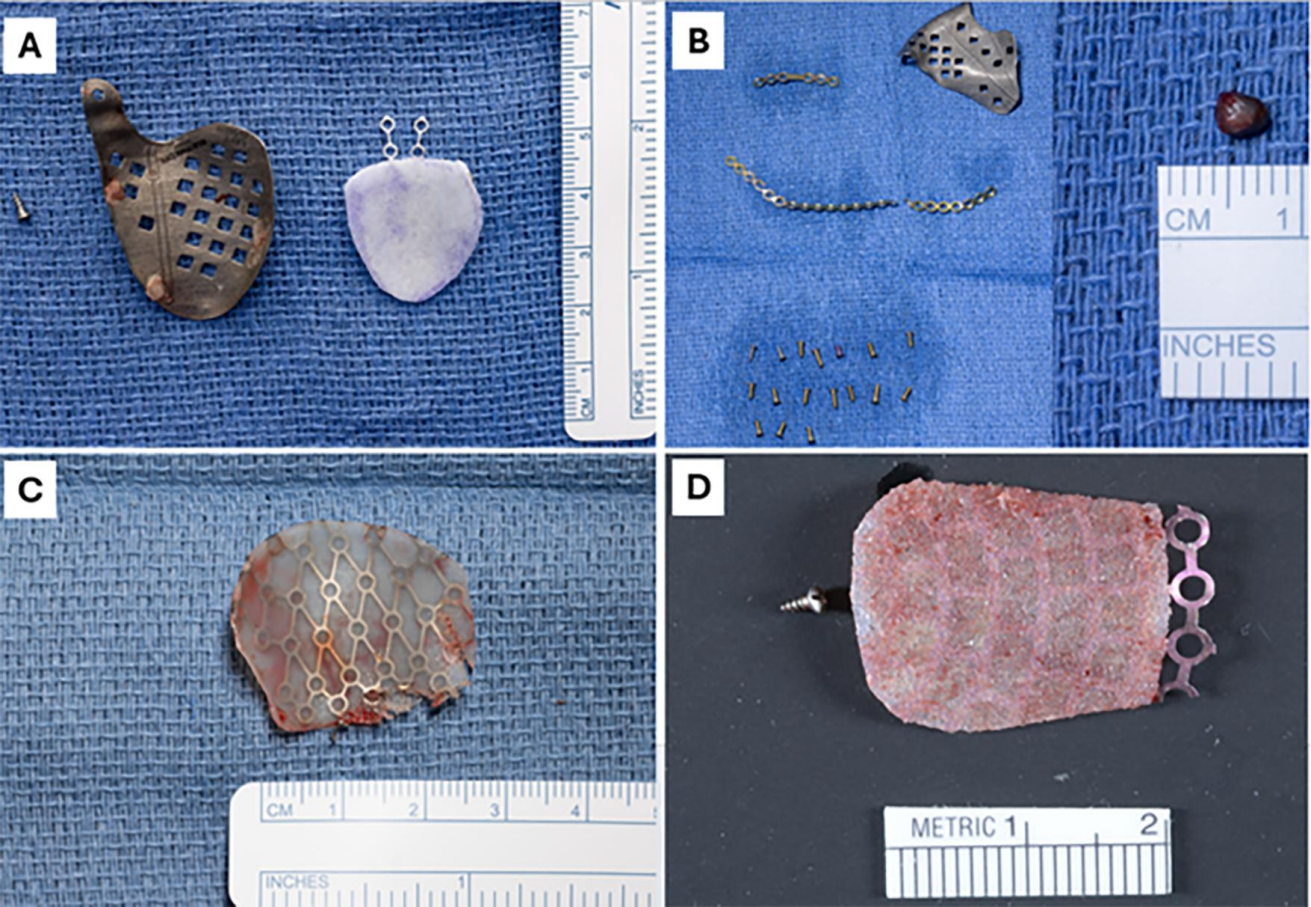
Explanted orbital implants, associated hardware, and retrieved specimens. **(A)** Removed orbital implant (left) shown alongside the new-custom MEDPOR® TITAN implant that was used for medial/floor orbital wall reconstruction (right). **(B)** Retrieved hardware components, including implant fragments, plate/chain segments, and multiple fixation screws, a small foreign body fragment. **(C)** An explanted MEDPOR® TITAN implant demonstrating surface fenestrations and adherent capsule. **(D)** Explanted orbital implant with the associated fixation screw.

**Figure 5:**
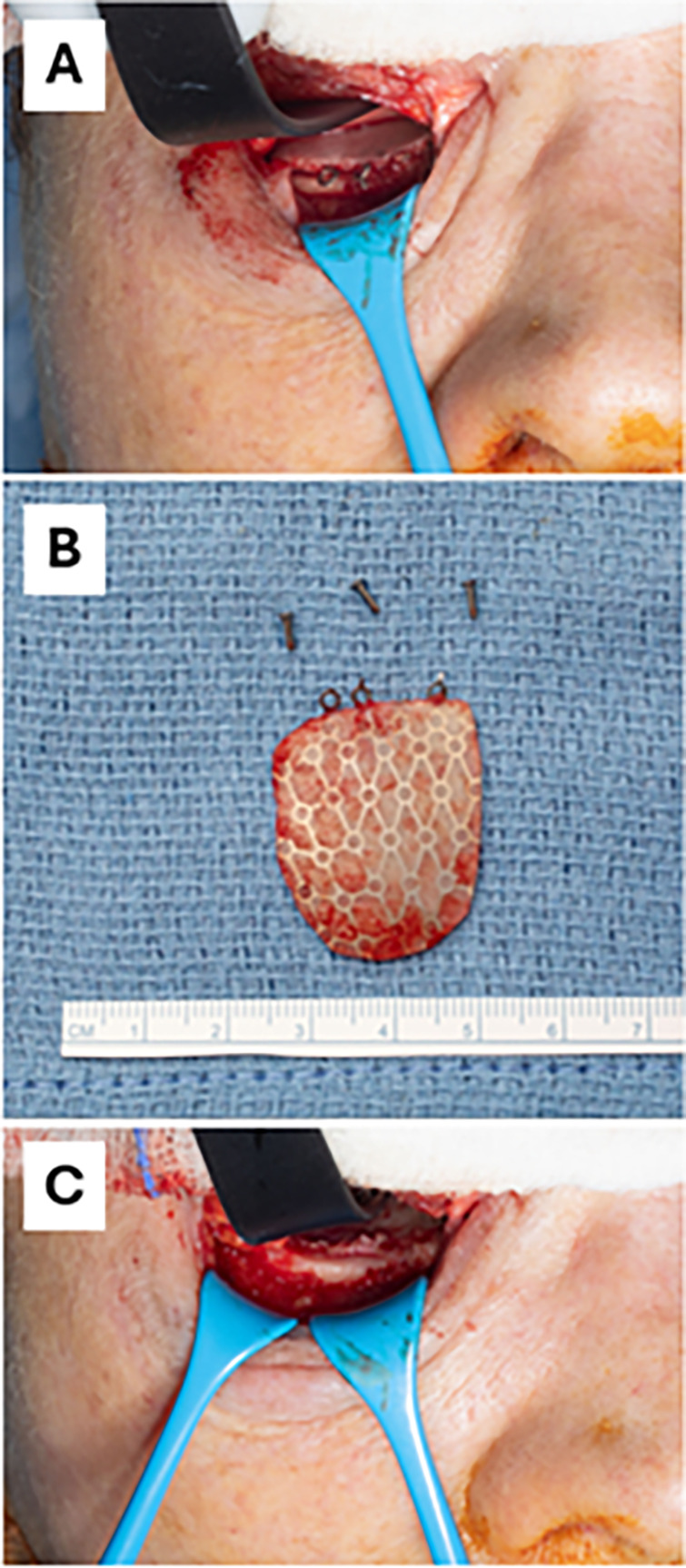
Intraoperative pictures demonstrating orbital floor implant removal via transconjunctival approach. **(A)** Inferior lid retraction with Desmarres retractor exposing the Medpor Titanium implant at the inferior orbital rim. **(B)** Removed implant (∼3.5 × 2.5 cm) with 3 fixation screws. **(C)** Post-removal view of the orbital floor.

Implants and their fixation screws were identified and removed (Figures 4, 5). In cases of dense fibrosis, inflammatory capsules, or heterotopic bone formation, adhesiolysis and piezo-assisted osteotomy were employed to safely mobilize the implant. Intraoperative navigation (Medtronic StealthStation™ navigation platform) and preoperative CT imaging were used selectively to confirm implant position, to assess proximity to critical structures, and guide safe dissection, particularly in cases involving intraconal extension or posterior orbital involvement.

Implant management strategies were individualized based on the dominant failure mechanism (Figure 9). In patients with inflammatory or infectious complications, complete implant removal with capsule excision and washout was performed. In cases of mechanical malposition or neuromuscular impingement without volume deficit and healthy capsule formation over the fracture, explantation alone was deemed adequate. Patients with orbital volume mismatch causing visible deformity, uncorrected medial wall defect, enophthalmos or hypoglobus underwent revision. This was done using semi-custom Porous Polyethylene Implants (MEDPOR Titan; © Stryker, Kalamazoo, MI, USA) contoured intraoperatively on 3D printed patient-specific anatomical models, or custom Polyetheretherketone (PEEK) implants manufactured based on mirror image VSP. Implants were secured with titanium screws, and positioning was confirmed visually, with intraoperative navigation and, when indicated, with intraoperative CT imaging. Additionally, if infection was suspected, cultures of the capsule were sent for microbiology. Dense fibrotic encapsulation was encountered in five patients, requiring extensive adhesiolysis.

In Cases 2 and 4, the implant had extended around the inferior oblique muscle, precluding direct removal without controlled myotomy. In Case 5, a sheet of heterotopic bone had fused over the lateral half of the implant, requiring piezo-assisted osteotomies prior to plate removal. Case 3 involved dense adhesion of the plate into the right nasal sidewall, necessitating division of the implant into two pieces for retrieval. Notably, implant contour problems can coexist with otherwise appropriate anteroposterior and mediolateral implant positioning. Excessive convexity may result in intraconal encroachment and paradoxical exophthalmos despite apparently satisfactory implant placement. Case 7 illustrated a milder version of this phenomenon, with a floor implant in an acceptable position but slightly elevated from the native orbital floor and associated with persistent inflammation. Intraoperative navigation (Medtronic StealthStation™; accuracy 0.7–0.8 mm), using a previously developed institutional FaceNav workflow (Sharaf et al., submitted), was employed in four cases (57.1%) to confirm implant position relative to critical structures in real time.

### Revision strategies

Complete explantation without PSIs was performed in four patients (57.1%). In each of these cases, either the fibrous capsule provided adequate structural support over the fracture and did not warrant additional implant placement at the time of revision. Intraoperative cultures were obtained in all infectious cases and forwarded to microbiology; notably, cultures from Case 6 showed no bacterial growth despite frank purulence, potentially reflecting prior antibiotic exposure. Secondary reconstruction with PSIs was performed in three patients (42.9%). Case 1 received a semi-custom MEDPOR Titan orbital floor and medial wall implant, contoured on a sterilized 3D-printed mirror image model and secured with two 4-mm titanium screws. Cases 2 and 3 received a custom-bent MEDPOR Titan, and PEEK implants contoured preoperatively on the 3D printed VSP model, respectively. In all cases, forced duction testing confirmed globe freedom from restriction at the conclusion of the procedure.

Closure was performed in layered fashion with absorbable sutures. The periosteum was reapproximated over the implant or orbital rim, followed by conjunctival closure. Lateral canthal reconstruction, eyelid repositioning, scar revision, and fistula excision were performed as needed by the oculoplastic team. Antibiotic ophthalmic ointment was applied at the conclusion of the procedure, and all patients tolerated outpatient surgery without intraoperative vision loss (Table 3).

**Table 3:** Surgical approach, implant management, and intraoperative findings.

Ca se	Follo w-up Durat ion (mont hs)	Anesthesia Type/Durati on	Operat ive time	Surgical Approach	VSP/3D Plann ing	Navigat ion (Stealth)	Implant(s) Removed	Periimplant Fluid	Intraoperative Findings	Estimated Blood Loss (mL)	Same-Day Additional Procedures
1	2	General, 2 h 22 min	1 h 34 min	Transconjunctival	No	No	Bare titanium plate (placed after prior silastic sheet removal, 2019)	None	Proximity to optic nerve at orbital apex; supraorbital/supratrochlear/inf erior trochlear vessels; screw possibly exposed to maxillary sinus	75	Medpor Titanium orbital floor & medial wall implant + 2 × 4 mm KLS mid-face screws
2	16.4	General, 4 h 27 min	2 h 49 min	Transconjunctiv al + transcaruncular + lateral canthotomy/cant holysis	Yes	Yes (0.8 mm accurac y)	Left ZM plate (infected)	Pus	Hardware infection; intraconal position with contact to extraocular muscles; inferior oblique in contact with old implant	25	Left hardware removal ZM plate (infection); fat grafting left orbit; custom PEEK implant placement
3	1.1	General, 8 h	5 h 46 min	Transoral (hemi LeFort 1); transfacial (glabellar/medial orbit scar); lateral brow incision; transconjunctiva l + transcaruncular + lateral canthotom	Yes	No	Left ZMF plate; right frontonasoma xillary plate; right lateral brow foreign body (3 mm black round object)	None	Extensive fibrosis of plate into right nasal sidewall; plate cut in 2 pieces for retrieval; dissection challenging due to fenestrations/undercuts/posteri or convexity of left orbital floor plate	75	Medpor Titan plate placement (left orbit reconstruct ion); screw fixation
4	8.4	General, 4 h 56 min	2 h 47 min	Transconjunctiv al + transcaruncular + lateral canthotomy/cant holysis	Yes	Yes (0.7 mm accurac y)	Right orbital floor/lateral wall implant (inadequate size)	None	Inferior oblique muscle released (plate extended on either side); dense fibrous tissue around superior/posterior medial wall near superior oblique; plate cut in pieces to facilitate removal	25	Dermal fat graft (3.5 × 1.2 cm) right lower eyelid (right buttock); orbicularis oculi flap suspension; Z-plasty right lateral canthal
											webbing (4 × 1 cm); right lateral canthopexy
5	5.4	General, 4 h	1 h 40 min	Previous tear trough/lower lid scar incision	Yes	Yes (0.7 mm accurac y)	Medpor Titan plate (left orbital medial wall)	Seros angui neous	Fluid-filled lesion; granulation tissue; newly formed bone sheet over lateral half of plate (required piezo osteotomies); heterotopic ossification. Pathology: pyogenic granuloma; dense connective & bone tissue with reactive changes; xanthelasma (left lower eyelid)	25	Xanthelas ma excision (2 × 1 cm elliptical); scar revision lower lid
6	2.2	General, 1 h 45 min	0 h 50 min	Left lower lid horizontal incision (7 mm from lash line) encompassing fistula tract	No	No	Left orbital floor Medpor titanium implant (infected)	Purul ent (sent for cultur e)	Fistula tract excised (1 × 1 × 0.5 cm); purulent material from capsule drained; single screw at orbital rim removed. Culture: no bacterial growth	5	Excision of fistula tract; multilayered wound repair
7	1.4	General 4 h 19 min	1 h 26 min	Transconjunctiv al + lateral canthotomy/cant holysis	Yes	Yes (0.8 mm accurac y)	Right orbital floor Medpor Titan implant (suturefixated with 3 sutures)	None	Healthy-appearing pseudocapsule around implant; minimal residual restriction on forced ductions (infraduction/abduction); internal capsule surface cauterized. No purulence or infection.	20	Internal capsule cauterizati on; anterior capsule rim excision

Histopathological findings were obtained for two cases; Case 5 specimens were diagnosed as pyogenic granuloma with dense connective and bone tissue showing reactive changes and a concurrent xanthelasma at the lower lid. Case 6 capsular material showed acute and chronic inflammation without organisms on Gram stain or culture.

### Postoperative outcomes

**Figure 6:**
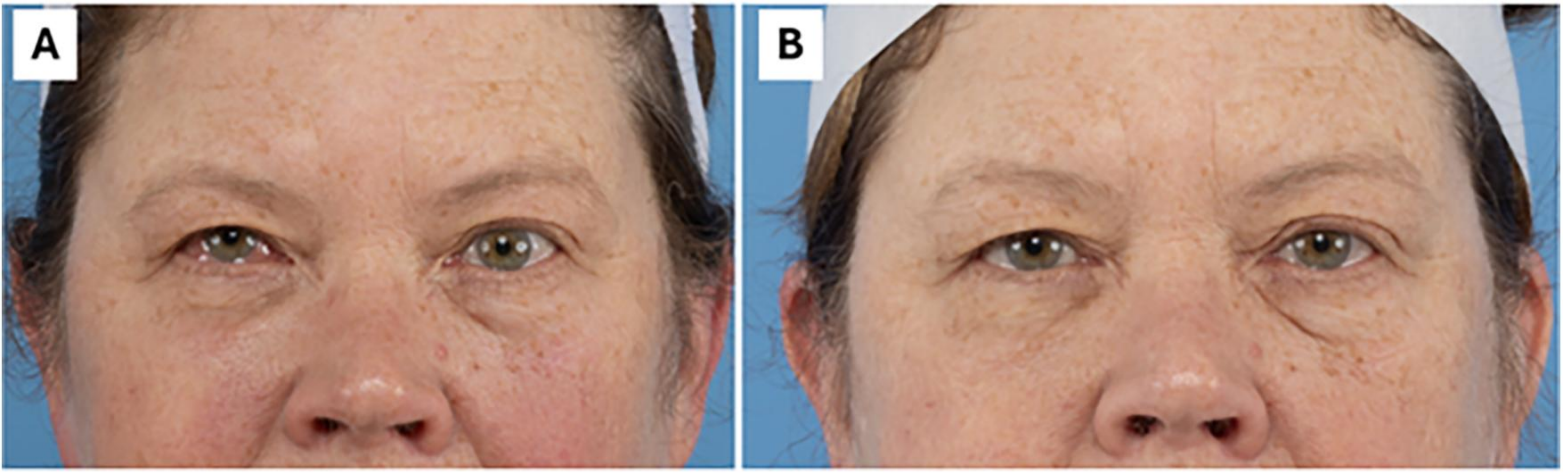
Improvement of strabismus after hardware removal. **(A)** Preoperative view showing left hypertropia, and **(B)** Postoperative result at 3-month follow-up. Note improved alignment of the eyes with persistent lower lid retraction. The patient had surgery for residual strabismus correction 5 months after orbital hardware removal.

**Figure 7:**
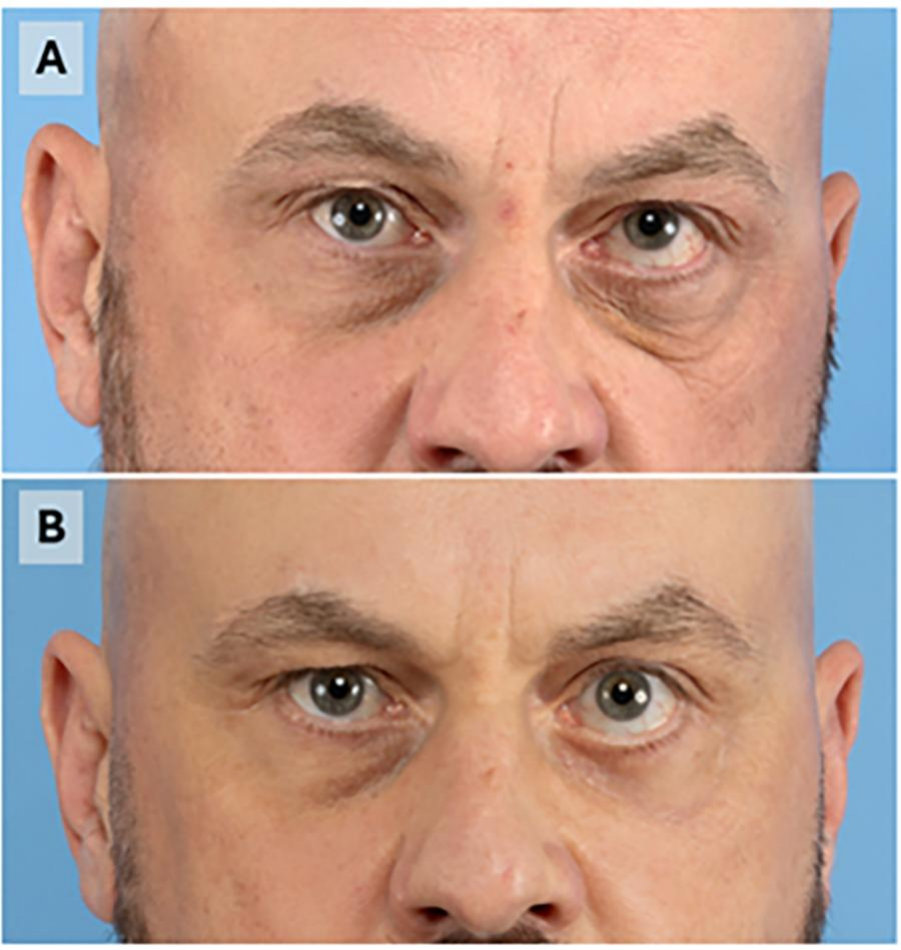
Ocular alignment improvement after hardware removal. **(A)** Preoperative view showing ocular misalignment with symptomatic diplopia. **(B)** Postoperative result at follow-up, showing improved alignment of the eyes.

Postoperative outcomes are summarized in Table 4. At a mean follow-up of approximately 612 months, most patients demonstrated meaningful clinical improvement: Diplopia improved or resolved in 4 of 5 affected patients (80%), consistent with prior reports demonstrating 85%–90% improvement rates (Figures 6, 7); globe position and enophthalmos improved in all patients undergoing implant removal or secondary reconstruction with PSIs. Likewise, pain and swelling improved in all symptomatic patients (100%). One patient (Case 5) demonstrated residual left hypertropia improved from 55 to 40 prism diopters following hardware removal alone, subsequently undergoing formal strabismus surgery (right superior oblique recession 5 mm with adjustable suture and left inferior rectus plication 5 mm). Case 7 retains symptomatic diplopia and is being followed in coordination with ophthalmology for anticipated strabismus intervention.

**Table 4:** Postoperative outcomes, complications, and additional procedures.

Case	Secondary Reconstruction Implant	Clinical Outcomes	Unfavorable Sequelae	Additional Surgeries
1	Medpor Titanium orbital floor & medial wall implant + 2 × 4 mm KLS screws	Pain/swelling improved	Binocular diplopia persists	—
2	Custom-made PEEK implant	Diplopia 80% improved; mild residual vertical diplopia	Left upper lid retraction with ocular exposure; left lower lid entropion with lash-corneal touch	One year later, the patient underwent bilateral upper eyelid blepharoplasty, brow lift, micro fat grafting to the left cheek and temple, dermal fat grafting to the left temple, and removal of hardware from the left lateral orbital rim, performed for improved cosmetic outcome. Approximately 6 months after the blepharoplasty and fat grafting, the patient underwent correction of left lower eyelid entropion with lash-corneal contact and
				correction of left upper eyelid retraction associated with ocular surface exposure.
3	Medpor Titan plate	Pain/swelling improved; diplopia improved	—	—
4	None (hardware removed; soft tissue reconstruction performed)	Diplopia persists (vertical + torsional on downgaze; superior rectus weakness)	Persistent diplopia; cicatricial ectropion; inducible strabismus with lateral and upward gaze	5 months later, repeat dermal fat graft right lower eyelid; orbicularis oculi flap suspension; Z-plasty; right lateral canthopexy. Note: upward lateral strabismus procedure discussed; ongoing follow-up planned
5	None	Left hypertropia reduced: 55 → 40 prism diopters post hardware removal	Residual motility deficit; left hypertropia	5 months later, strabismus procedure was performed right superior oblique recession 5 mm (adjustable) + left inferior rectus plication 5 mm after adjustment
6	None (hardware removed; no reconstruction)	Infection resolved	—	—
7	None (hardware removed; no reconstruction)	Minimal residual restriction on forced ductions postoperatively	Persistent diplopia; likely requires strabismus surgery	Strabismus procedure discussed; ongoing follow-up planned

Three patients required additional procedures for aesthetic purposes beyond the index revision surgery to address residual eyelid malposition or soft tissue deficiency. These included upper eyelid blepharoplasty, brow lift, fat grafting, and correction of lower eyelid entropion with lash-corneal touch in Case 2; repeat dermal fat grafting, Z-plasty, and canthopexy in Case 4; and ongoing strabismus management in Cases 5 and 7. None of the patients in our series developed clinically apparent nasolacrimal duct obstruction after revision surgery, experienced vision- or life-threatening complications, or required urgent reoperation.

## Discussion

**Figure 8:**
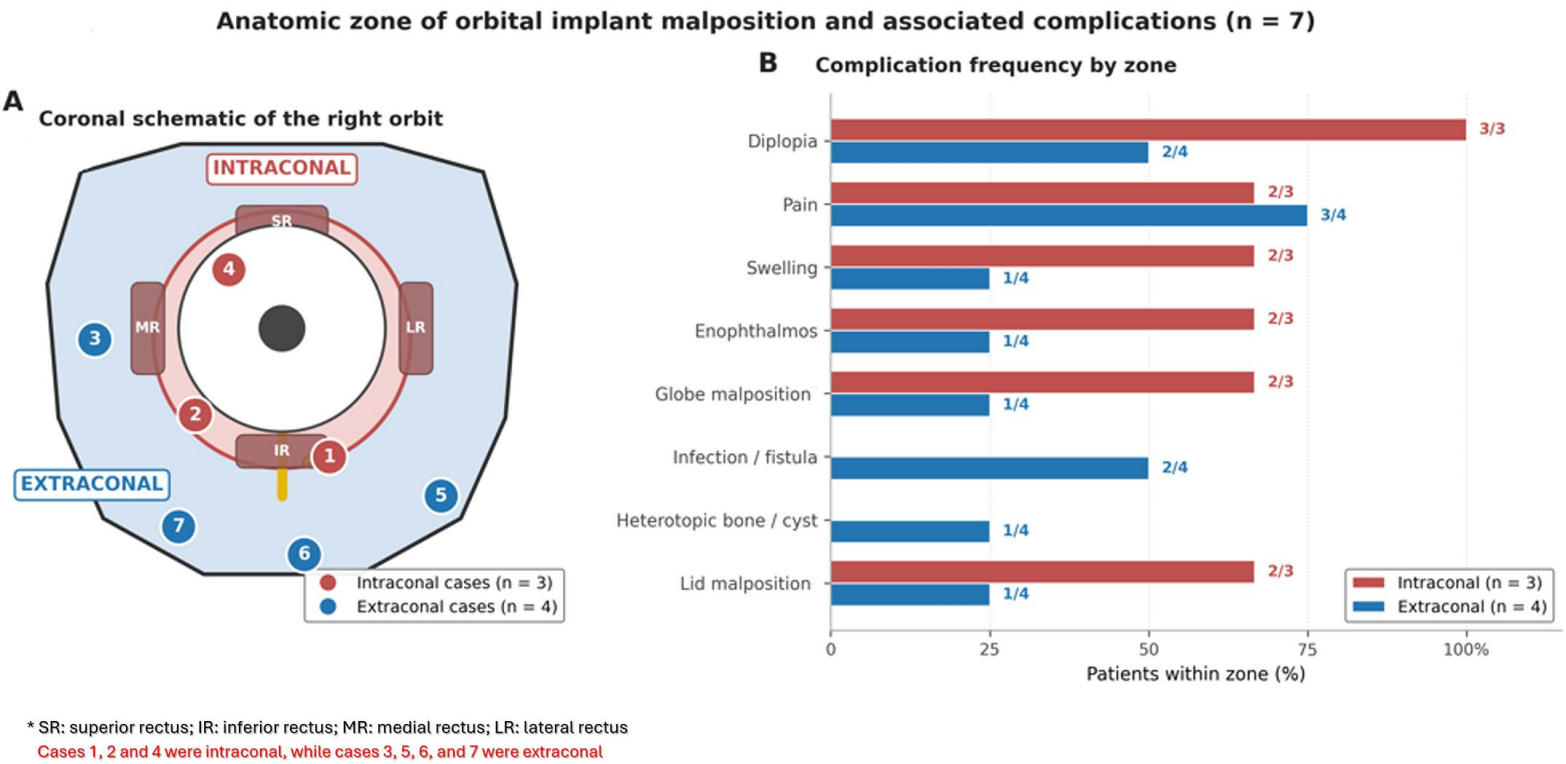
Anatomic zones of orbital implant malposition and associated presenting complaints (cases 1–7). **(A)** Coronal schematic illustration of the right orbit and the affected zone by the malpositioned implant. **(B)** Presented Complication Frequency by zone.

**Figure 9:**
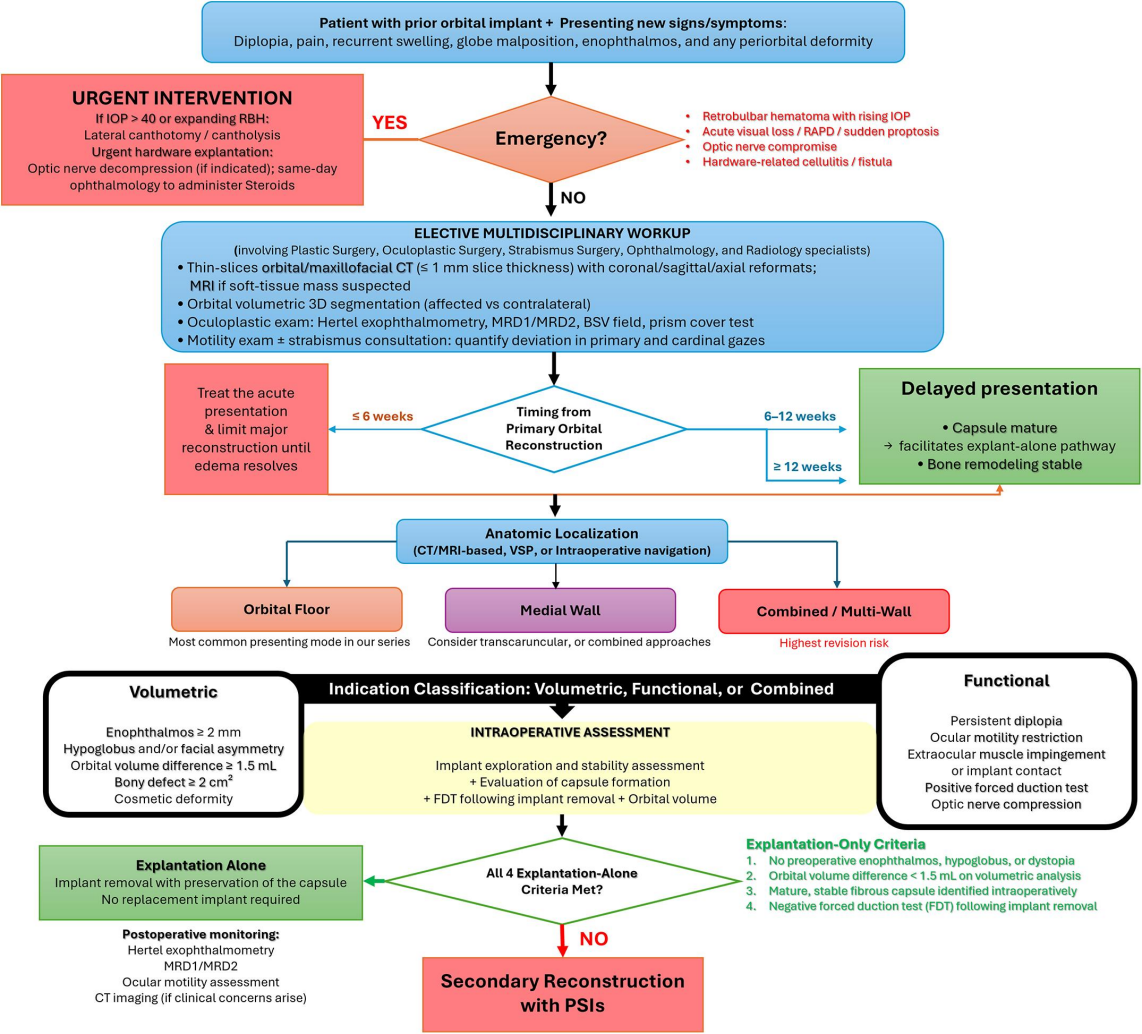
Proposed clinical decision-making framework for revision surgery in patients with malpositioned orbital implants, integrating symptoms, imaging findings, and surgical strategy.

This retrospective series offers a granular, multidisciplinary perspective on the management of malpositioned orbital implants, a challenging clinical entity that has received limited systematic attention despite its significant functional consequences. Across seven consecutive cases, we identify four dominant failure mechanisms (Figure 8): (1) Mechanical malposition or soft-tissue violation (most common), (2) muscular or neurovascular impingement, (3) inflammatory or infectious reaction, and (4) volume deficiency or orbital deformity. Our findings emphasize that implant failure is rarely unidimensional and often results from a combination of mechanical malposition, soft-tissue violation, neurovascular impingement, and inflammatory responses. Our clinical decision-making framework for revision surgery in patients with malpositioned orbital implants, integrating symptoms, imaging findings, and surgical strategy are shown in Figure 9.

Each surgical approach to the orbit carries its own profile of acceptable trade-offs. Pre-septal transconjunctival exposure preserves orbital fat compartments but offers a more constrained working corridor than the post-septal route, which provides wider exposure at the cost of greater fat-pad disruption and potential post-traumatic fat atrophy that contributes to delayed enophthalmos (11–13). Sub-periosteal dissection for exposure of the medial and inferior walls, even when performed correctly, can fail to re-approximate periorbita over the implant, leaving the implant exposed to direct soft-tissue contact and contributing to capsule formation (14). These approach-related variables, combined with extraconal fat atrophy from the index trauma itself (15), should be considered when counseling patients about expected outcomes after revision.

### Implant malposition and concordance with clinical symptoms

A key observation is the strong concordance between clinical symptoms and radiographic findings (1, 6, 16–18). In all patients presenting with diplopia, preoperative imaging demonstrated either implant contact with extraocular musculature or intraconal extension, supporting a direct relationship between implant position and functional impairment. These findings are consistent with prior reports describing extraocular muscle tethering and orbital apex crowding as contributors to postoperative diplopia (7, 8). Our series corroborates these findings and builds upon them by demonstrating that intraconal extension, where the implant protrudes beyond the equator of the globe into the muscle cone, is a particularly important predictor of persistent diplopia (12, 19). In rare but catastrophic cases, implant migration can result in intraocular penetration with open globe injury, as reported by De Clerck et al. and Chandra et al., underscoring the importance of early recognition and intervention (5, 20). In addition to intraorbital complications, malpositioned orbital implants have also been reported to cause mechanical obstruction of the ostiomeatal complex, resulting in maxillary sinus atelectasis and enophthalmos (6, 13, 21).

Hsu et al. demonstrated that both trauma and surgical manipulation can compromise ocular motor nerve function, resulting in neurogenic causes of diplopia beyond the traditionally recognized myogenic causes such as failure to relieve entrapment (22). In our series, Cases 2 and 4 demonstrated implant extension around the inferior oblique muscle, precluding direct removal without controlled myotomy — illustrating how implant malposition can create secondary muscle entrapment distinct from the initial traumatic injury. Park et al. evaluated outcomes of inferomedial blowout fracture repair with and without inferior oblique reattachment after detachment, finding that patients with preoperative strabismus and extraocular motility limitation had increased risk of postoperative complications regardless of reattachment technique (23).

### Explantation alone versus secondary reconstruction

Importantly, complete implant removal without replacement resulted in satisfactory symptom improvement in half of the cohort. This challenges the assumption that revision of orbital surgery requires implant replacement. In cases where symptoms were driven by inflammatory reaction, protrusion into soft-tissue, or neurovascular impingement without significant volume deficiency, explantation alone was sufficient. Conversely, patients with implant undersizing, asymmetry, or persistent enophthalmos benefited from secondary reconstruction or implant replacement. Based on these findings, we underscore the importance of distinguishing implant-driven from muscle-driven diplopia when planning revision strategy, as the former may respond to explantation alone while the latter may require staged strabismus surgery (22–24). Orthoptic examination and correlation of motility restriction patterns with radiographic findings can support this diagnostic decision. In addition, incorporation of patient-specific technologic advancements such as 3D VSP, mirror-image analysis, and intraoperative navigation further enhances preoperative decision-making, implant design, and surgical precision (25, 26).

For patients requiring secondary reconstruction, patient-specific implants (PSIs) offer significant advantages. Schreurs et al. reported a cohort of 22 patients treated with PSIs for secondary post-traumatic orbital reconstruction, demonstrating significant improvements in diplopia, and hypoglobus, with accurate implant positioning confirmed by quantitative analysis (13). A systematic review of 1,222 subjects confirmed that PSIs achieve significantly reduced persistent enophthalmos (7.3% vs. 18.2%), lower persistent diplopia (11.7% vs. 30.1%), and reduced revision rates (5.9% vs. 13.7%) compared to conventional reconstruction (27). In our series, secondary reconstruction was performed in three patients (42.9%) using semi-custom MEDPOR Titan implants contoured on 3D-printed mirror-image models or custom PEEK implants, with intraoperative navigation confirming accurate placement.

### Late complications: cystic lesions and hemorrhage

An important finding from our series and the broader literature is the recognition of late-onset complications that may present years to decades after initial implantation. Kohyama et al. demonstrated that silicone implants used for orbital wall fractures were “never stabilized” during long-term follow-up, with overall complication rates increasing from 5/47 to 13/47 and implant removal rates increasing from 0/47 to 10/47 over a mean follow-up time of 83.1 months (28). Most late complications were caused by cystic lesions, and implant removal alone was sufficient and safe as the initial intervention. Similarly, “chocolate cysts” or “hemorrhagic cystic lesions”, associated with porous polyethylene implants, have been reported 3 to 9 years after orbital fracture repair (11). Particularly, the historical fenestrated titanium mesh, which was seen in one case in our series, may permit ingrowth of adjacent sinonasal epithelium, creating a substrate for inclusion cyst formation (4). In addition, perforations may trap serous fluid or chronic hemorrhage, resulting in progressive cystic expansion (4). Although this reported late orbital hemorrhage around alloplastic orbital floor implant is a rare complication, it should be considered in patients presenting with delayed orbital symptoms. Suller et al. described three patients with chocolate cysts presenting with mass effect symptoms, all successfully managed by implant removal and cyst evacuation (14). Epithelial cysts represent another distinct late complication, with Su et al. reporting a median presentation of 8 years (range 15 months to 31 years) after implantation in their systematic review (29).

Based on these observations, we propose a practical decision framework for revision orbital surgery (Figure 9). Patients should first be stratified by dominant symptom (functional vs. volumetric). CT imaging should then be assessed for failure patterns, guiding the choice between implant removal alone, or secondary reconstruction with customized implants. If there is no volumetric defect, but intraoperative exploration shows absence of a stable capsule over the fracture, simple replacement with a standard implant placed in correct position should be performed. As recently described by Shoji et al. (6), the malpositioned orbital implant may also manifest with secondary sinus obstruction and maxillary atelectasis. This observation underscores the importance of comprehensive radiographic evaluation extending beyond the orbital soft tissues to include adjacent sinus anatomy. Incorporating this broader assessment into surgical planning may optimize clinical outcomes while avoiding unnecessary implant replacement. This approach is supported by our previously reported decade-long institutional experience with VSP and 3D printing in facial trauma, which demonstrated enhanced preoperative accuracy and increased intraoperative confidence (26).

### Role of navigation-guided surgery

Intraoperative CT allows real-time assessment of fracture reduction and immediate implant revision, especially at the posterior ledge of the orbit (30). Chu et al. demonstrated that the surgical navigation system with intraoperative 3D C-arm CT effectively improved the accuracy of zygomatico-orbital fracture reconstruction and decreased implant adjustment times, with no significant difference between postoperative results and preoperative virtual planning (30). Intraoperative navigation can help confirm appropriate posterior implant positioning while maintaining a safe distance from the orbital apex and optic nerve. In our series, intraoperative navigation (Medtronic StealthStation; accuracy 0.7–0.8 mm) was employed in four cases (57.1%) to confirm implant position relative to critical structures in real time. Thus, navigation is particularly valuable in the revision setting, where distorted anatomy and dense fibrosis make accurate implant placement especially challenging.

### Contribution of Fat atrophy and soft tissue changes to enophthalmos

The posttraumatic enophthalmos is multifactorial and cannot be attributed solely to implant malposition. Nevertheless, accurate restoration of posterior orbital support is critical in primary or secondary reconstruction. Insufficient posterior implant extension may result in implant descent into the maxillary sinus with persistent orbital volume expansion, hypoglobus, or enophthalmos, whereas excessive posterior extension increases the risk of injury to the neurovascular structures crossing through the superior and inferior orbital fissures and the optic nerve (12, 15). Similar principles apply to medial wall reconstruction, where the sphenoid body may represent the only stable posterior support structure when the lamina papyracea is absent. Additionally, extraconal fat atrophy, whether from the initial trauma, surgical approach, or chronic peri-implant inflammatory changes, contributes to loss of globe projection. Manson et al. demonstrated that the principal mechanism of posttraumatic enophthalmos involves posterior displacement and reshaping of orbital soft tissue from a modified cone to a sphere under the influence of gravity and scar contracture, rather than fat volume loss (15). A meta-analysis of 25 studies (648 patients) confirmed that bony orbital volume expansion accounts for approximately 50% of posttraumatic enophthalmos (31). Choi et al. further showed that herniated fat volume in inferior orbital wall fractures was the most significant predictor of late-onset enophthalmos (11). These soft tissue changes may confound the assessment of implant-related enophthalmos and must be considered when determining whether secondary reconstruction is indicated. In our series, patients with residual enophthalmos (Cases 1, 2, and 4) underwent secondary reconstruction with volume augmentation, while those without significant volume deficit were managed with explantation alone.

Limitations of this study include its retrospective design, small sample size, and single-center experience. Additionally, 5 of 7 index repairs were performed at outside institutions, which limited the granularity of historical approach data. Multidisciplinary teams and advanced patient-specific techniques may not be available at other practices. Symptom reporting was subjective, and follow-up duration varied. Nonetheless, the collaboration of surgeons with distinct areas of expertise, detailed imaging review, well-defined operative findings, and meticulous outcome documentation provide clinically meaningful insights into a challenging reconstructive scenario.

## Conclusion

Malpositioned orbital implants represent a complex cause of postoperative morbidity that requires individualized evaluation and management. Careful correlation of patient-reported symptoms with radiographic failure patterns is essential in selecting the appropriate revision strategy. In this series, tailored surgical management—including implant removal, replacement, or secondary reconstruction—resulted in meaningful improvement for most patients. Recognition of dominant failure mechanisms may assist surgeons in optimizing outcomes and avoiding unnecessary implant replacement. Incorporation of 3D VSP, mirror-image analysis, and intraoperative navigation (10) further enhances preoperative decision-making, implant design, and surgical precision.

## Data Availability

The raw data supporting the conclusions of this article will be made available by the authors, without undue reservation.

## Supplementary material

Supplementary Table S1 Preoperative and postoperative oculoplastic measurements, eyelid parameters, and motility findings for all patients undergoing implant removal (Explantation Alone) with or without secondary orbital reconstruction. Hertel exophthalmometry, margin reflex distance (MRD1 and MRD2), globe position, ocular motility, diplopia characteristics, and eyelid malposition are reported at the most recent available preoperative and postoperative evaluations.
