# Supplementary material for: Malpositioned orbital implants: indications for implant removal and secondary reconstructive techniques
Source: Front Surg. 2026 Jul 17;13:1878546. doi: 10.3389/fsurg.2026.1878546 (PMC13424244; doi:10.3389/fsurg.2026.1878546)
Supplement: Supplementary Table S1 — Preoperative and postoperative oculoplastic measurements, eyelid parameters, and motility findings for all patients undergoing implant removal (Explantation Alone) with or without secondary orbital reconstruction. Hertel exophthalmometry, margin reflex distance (MRD1 and MRD2), globe position, ocular motility, diplopia characteristics, and eyelid malposition are reported at the most recent available preoperative and postoperative evaluations. [file Table1.docx]

**Supplemental Table 1**

| Case | Time-point | Hertel (R / L × base, mm) | MRD1 (R / L, mm) | MRD2 (R / L, mm) | Motility, Globe and Eyelid Malposition |
| --- | --- | --- | --- | --- | --- |
| 1 | **Pre** | 16 / 15 × 97 | 4 / 4 | 5 / 6 | Mild L hypertropia on upgaze; diplopia-free in primary gaze |
| 1 | **Post** | NR | NR | NR | _ |
| 2 | **Pre** | 19 / 15 × 101 | 5 / 4 | NR | L enophthalmos + hypoglobus; L hypotropia worse in upgaze/L gaze |
| 2 | **Post** | 18 / 17 × 101 | 4 / 5 | NR | Residual vertical binocular diplopia; L upper-lid central peak; intermittent lower-lid entropion |
| 3 | **Pre** | 20 / 15 × 107 | 3.5 / 2 | NR | 5 mm L enophthalmos |
| 3 | **Post** | 20 / 15 × 107 | 5 / 0 | 5 / 3 | No diplopia; hollow L sulcus without crease |
| 4 | **Pre** | 15 / 14 × 90 | 3 / 3 | 6 / 6 | Vertical diplopia primary gaze; 50% upgaze restriction in adduction; R supraduction restriction |
| 4 | **Post** | NR (canthal edema) | 4 / 4 | 7 / 6 | Inducible strabismus on lateral/upward gaze; bilateral superior sulcus hollowing |
| 5 | **Pre** | 16 / 19 × 100 | 4 / 4 | 6 / 6 | L hypertropia worse in downgaze (55 PD); left head-turn AHP; ET |
| 5 | **Post** | 15 / 17 × 95 | NR | 6 / 8 | L hypertropia improved (40 PD); residual diplopia worse looking down |
| 6 | **Pre** | NR | NR | NR | Presenting complaint infectious (cellulitis + fistula), not motility |
| 6 | **Post** | 14 / 15 × 103 | NR | 8 (L) | Full EOM; no diplopia; mild L lower-lid retraction; no ectropion |
| 7 | **Pre** | 19 / 19.5 × 106 | 3 / 4 | 5 / 5 | Constant binocular diplopia; FDT no restriction in nasal/lateral-temporal/inferior gaze |
| 7 | **Post** | 18 / 19 × 95 | 3 / 3 | 6 / 6 | Full EOM at early follow-up |
| *NR = not recorded; PD = prism diopters; FDT = forced duction test; AHP = abnormal head posture; EOM = extraocular motility; ET = esotropia.* | | | | | |
| *Hertel format: right eye / left eye × base distance (mm).* | | | | | |

**Supplemental Table 1.** Preoperative and postoperative oculoplastic measurements, eyelid parameters, and motility findings for all patients undergoing implant removal (Explantation Alone) with or without secondary orbital reconstruction. Hertel exophthalmometry, margin reflex distance (MRD1 and MRD2), globe position, ocular motility, diplopia characteristics, and eyelid malposition are reported at the most recent available preoperative and postoperative evaluations.
